# Neonatal Sepsis: The Impact of Carbapenem-Resistant and Hypervirulent *Klebsiella pneumoniae*

**DOI:** 10.3389/fmed.2021.634349

**Published:** 2021-06-11

**Authors:** Subhankar Mukherjee, Shravani Mitra, Shanta Dutta, Sulagna Basu

**Affiliations:** Division of Bacteriology, Indian Council of Medical Research (ICMR)-National Institute of Cholera and Enteric Diseases, Kolkata, India

**Keywords:** neonatal sepsis, *Klebsiella pneumoniae*, carbapenem resistance, hypervirulence, CR-hvKP, antibiotics, treatment, lower-middle-income countries

## Abstract

The convergence of a vulnerable population and a notorious pathogen is devastating, as seen in the case of sepsis occurring during the first 28 days of life (neonatal period). Sepsis leads to mortality, particularly in low-income countries (LICs) and lower-middle-income countries (LMICs). *Klebsiella pneumoniae*, an opportunistic pathogen is a leading cause of neonatal sepsis. The success of *K. pneumoniae* as a pathogen can be attributed to its multidrug-resistance and hypervirulent-pathotype. Though the WHO still recommends ampicillin and gentamicin for the treatment of neonatal sepsis, *K. pneumoniae* is rapidly becoming untreatable in this susceptible population. With escalating rates of cephalosporin use in health-care settings, the increasing dependency on carbapenems, a “last resort antibiotic,” has led to the emergence of carbapenem-resistant *K. pneumoniae* (CRKP). CRKP is reported from around the world causing outbreaks of neonatal infections. Carbapenem resistance in CRKP is largely mediated by highly transmissible plasmid-encoded carbapenemase enzymes, including KPC, NDM, and OXA-48-like enzymes. Further, the emergence of a more invasive and highly pathogenic hypervirulent *K. pneumoniae* (hvKP) pathotype in the clinical context poses an additional challenge to the clinicians. The deadly package of resistance and virulence has already limited therapeutic options in neonates with a compromised defense system. Although there are reports of CRKP infections, a review on neonatal sepsis due to CRKP/ hvKP is scarce. Here, we discuss the current understanding of neonatal sepsis with a focus on the global impact of the CRKP, provide a perspective regarding the possible acquisition and transmission of the CRKP and/or hvKP in neonates, and present strategies to effectively identify and combat these organisms.

## Introduction

The infiltration of sterile regions of the body with microorganisms and the manifestation of a reaction to it thereof, is known as sepsis. When this occurs within the first 28 days of life consequences can be dire, as the newborns first encounter a world of pathogens. Their immune system has not yet developed to equip them in this battle. Thus, the total number of neonatal deaths due to sepsis is a staggering half-million per year (1, 2).

The pathogens that a neonate encounters are essentially present everywhere, starting with the birth canal, the crib, the hands of the nurse, or even the nasogastric tube. For the ones who are premature or low-birth-weight and require prolonged hospitalization or life support systems, the chances of infection are very high (3). In low-income countries (LICs) and lower-middle-income countries (LMICs), where nearly all resources are inadequate, breaches in care or infection control can lead to sepsis.

If one had to name an organism that readily becomes resistant to antibiotics, can harbor numerous plasmids, can survive in the environment & within the human gut, and is a dread in the neonatal intensive care units (NICU) in LMICs, it would be none other than *Klebsiella pneumoniae* (4, 5). This organism has increasingly shown various facets of a successful pathogen. Resistance to several antibiotics at a low fitness cost makes it capable of causing outbreaks in neonatal units. *K. pneumoniae* is resistant to a repertoire of antibiotics. Resistance to carbapenems, considered as the “last resort” against serious infections caused by Gram-negative bacilli, has limited therapeutic options immensely. As options of treatment were slowly failing, resistance to carbapenems was a cul-de-sac, because carbapenem-resistant *K. pneumoniae* (CRKP) are also resistant to several other antibiotics. Carbapenem-resistant genes are frequently harbored on plasmids that can spread from one species to the other. These resistance genes code for enzymes that efficiently hydrolyze carbapenems and all other β-lactam antibiotics. Further, most are also resilient against inhibition by the commercially viable β-lactamase inhibitors. In addition to carbapenem resistance, *K. pneumoniae* also had another ace up its sleeve, hypervirulence. Hypervirulent *K. pneumoniae* (hvKP) possess features that arm them to evade the host immune system to cause infections in immunocompetent hosts. hvKP are invasive and can disseminate to multiple sites.

With the World Health Organization (WHO) guidelines of treatment inadequately poised to tackle CRKP and hvKP in the neonatal population, this review presents different aspects of CRKP and hvKP and their impact on the newborn.

## Neonatal Sepsis: A Disease That Can't Be Ignored

According to the “Levels and Trends in Child Mortality report, 2019” the estimated global rate of neonatal deaths was 17 per 1,000 live births, and worldwide ~6,700 neonates died each day in 2019 (6). A notable disparity exists between high-income countries (HICs) and LMICs in terms of the rate of neonatal death (1, 6). Eighty percent of the entire global burden was from two regions: Sub-Saharan Africa (42%) and Central and Southern Asia (37%). In both these regions, the neonatal mortality rate is around 24 deaths per 1,000 live births whereas in North America and Europe the rate is three deaths per 1,000 live births (6). Conditions related to infections such as sepsis and pneumonia as well as conditions not related to infections such as preterm birth complications, intrapartum-related events (e.g., birth asphyxia), and congenital anomalies are the predominant causes of neonatal death (1).

Sepsis is a dysregulated host response to systemic infections leading to shock and multi-organ dysfunction (7, 8) and may or may not be associated with a positive blood culture (9). When a pathogen can be isolated from the blood or cerebrospinal fluid of a neonate (a child within 28 days from birth) with noticeable hemodynamic changes, it is defined as neonatal septicemia (3). Overall, the rate of neonatal sepsis varies between 6.5 and 38 per 1,000 live births (hospital born only) in LMICs with bloodstream infections (BSIs) ranging between 1.7 and 33 per 1,000 live births (10). These rates are 3–20 times higher than the rates of the industrialized countries which ranges between 1 and 5 per 1,000 live births (4). The neonatal sepsis rates from underdeveloped countries are not exactly represented in the above figures because in these countries many children are born at home. In the present COVID-19 situation, hospitalization for delivery has decreased further (11). Conversion of health management facilities to COVID-19 hospitals or shutting down of medical facilities due to COVID-19 spread has caused non-availability of proper treatment to all other critical life-threatening conditions (12, 13), including neonatal care (11, 14, 15).

In the most widely accepted notion, if sepsis is manifested within 72 h of life, it is defined as Early-onset sepsis (EOS), in which case, infections are conventionally thought to be transmitted from the mother. Any sepsis presenting after 72 h of life is defined as Late-onset sepsis (LOS) and the infection in such cases is thought to be hospital or community-acquired (3, 8). Premature and low-birth-weight neonates are more susceptible to infections caused by microorganisms and thus, for low-birth-weight neonates, every infection should be considered as hospital-acquired (3). In a recent study, a significant difference observed between EOS and LOS was associated with gestational age, as premature neonates showed higher rates of LOS and it is well-known that they are at higher risk of exposure to nosocomial infections as these neonates require longer hospital stays, central venous access, and often mechanical ventilation (16). In the same study, it was also noted that birth by cesarean section was more associated with LOS than birth by normal vaginal delivery (16). In LICs and LMICs, EOS and LOS cannot be properly distinguished. Due to poor hygienic practices in labor rooms and nurseries, every infection regardless of the time of onset can be hospital-acquired (10).

The disparity between LMICs and HICs is also reflected in the etiological agents causing the infections. In LMICs like India or Jordan, the causative agents of EOS are similar to causative agents of LOS (17–19). Studies show that Gram-negative bacteria, mainly *K. pneumoniae* and *Acinetobacter baumannii* play a pivotal role in causing neonatal septicemia along with *Escherichia coli* and Gram-positive *Staphylococcus aureus* in resource-poor settings (17–21). Whereas, in developed countries, group B *Streptococcus, E. coli*, and *S. aureus* are the major pathogens causing neonatal septicemia (3, 8).

## The Bug

*Klebsiella pneumoniae* is a Gram-negative, non-motile, and usually encapsulated bacillus of the Enterobacteriaceae family. This organism is omnipresent in the environment; *K. pneumoniae* is found in the soil, water, plants, and sewage. *K. pneumoniae* is also a part of the microbiome of the nasopharynx and gastrointestinal (GI) tract of healthy human beings (22, 23). It is an opportunistic pathogen and causes both hospital-acquired and community-acquired infections (24). In hospitals, *K. pneumoniae* causes both endemic infections and outbreaks of epidemic strains; chances of acquisition of *K. pneumoniae* in nasopharynx, GI tract, and skin increases with longer hospital stays and use of invasive devices (22). *K. pneumoniae* is one of the most predominant pathogens isolated from the intensive care units (ICUs) (23) and causes infections such as bacteremia, respiratory tract infections, urinary tract infection (UTI), invasive liver abscesses, endophthalmitis, and endocarditis (22, 25). Its capability of biofilm formation in the catheter enables it to cause catheter-associated UTI (26).

*K. pneumoniae* is also the predominant causative pathogen of neonatal sepsis (19, 27–30). Often a localized infection or colonization of the urinary tract, GI tract, or respiratory tract disseminates into the blood and leads to sepsis (26). The role of *K. pneumoniae* in causing neonatal sepsis is discussed later in detail.

Typing of strains is an integral part of epidemiological studies and presently multi-locus sequence typing (MLST), a method of distinguishing strains based on DNA sequences of internal fragments of multiple house-keeping genes, is prevalent. In *K. pneumoniae*, MLST is based on seven conserved housekeeping genes (*gapA, infB, mdh, pgi, phoE, rpoB*, and *tonB*) (31). The extensive drug-resistant (XDR) epidemic clones of *K. pneumoniae* are ST11, ST14, ST15, ST17, ST37, ST101, ST147, ST258, ST512, and these are reviewed elsewhere (32). *K. pneumoniae* can also be serotyped based on its capsular antigens (78 K antigens) (33).

Virulence of *K. pneumoniae* is essentially linked with its capsule which serves a dual purpose in the cell: it protects the cell from phagocytosis mediated by polymorpho-nuclear granulocytes and serum resistance by inactivation of one of the complement components (C3B). The pili or fimbriae is another component of the bacterial cell that helps in pathogenicity by mediating the adhesion of the pathogen to the mucosal layer and/or epithelial cells of the lower urinary tract, respiratory tract, and GI tract. Type 1 pili mediate adherence and then colonization of urinary and respiratory tract. Mannose-resistant Klebsiella-like hemagglutinin (MR/K-HA), a Type 3 pili, helps in the adhesion to Bowman's capsule, renal vessels, and tubular basal membranes of the human kidney (22). *K. pneumoniae* often possess large virulence plasmids (pLVPK) which harbor *rmpA, rmpA2*, and aerobactin biosysnthesis genes (34). With the possession of such virulence traits such as hypercapsule production, aerobactin and yersiniabactin synthesis, the bacterial cell becomes hypervirulent (34, 35).

For more than two decades, the increase in septicemia and meningitis in newborns caused by *Klebsiella* has been a matter of concern (22). Acquired resistance to critical antibiotics and hypervirulence in this fast-evolving pathogen is bound to make the existing scenario even more unmanageable.

## The Resistance of the Bug to the Drugs

**Figure 1:**
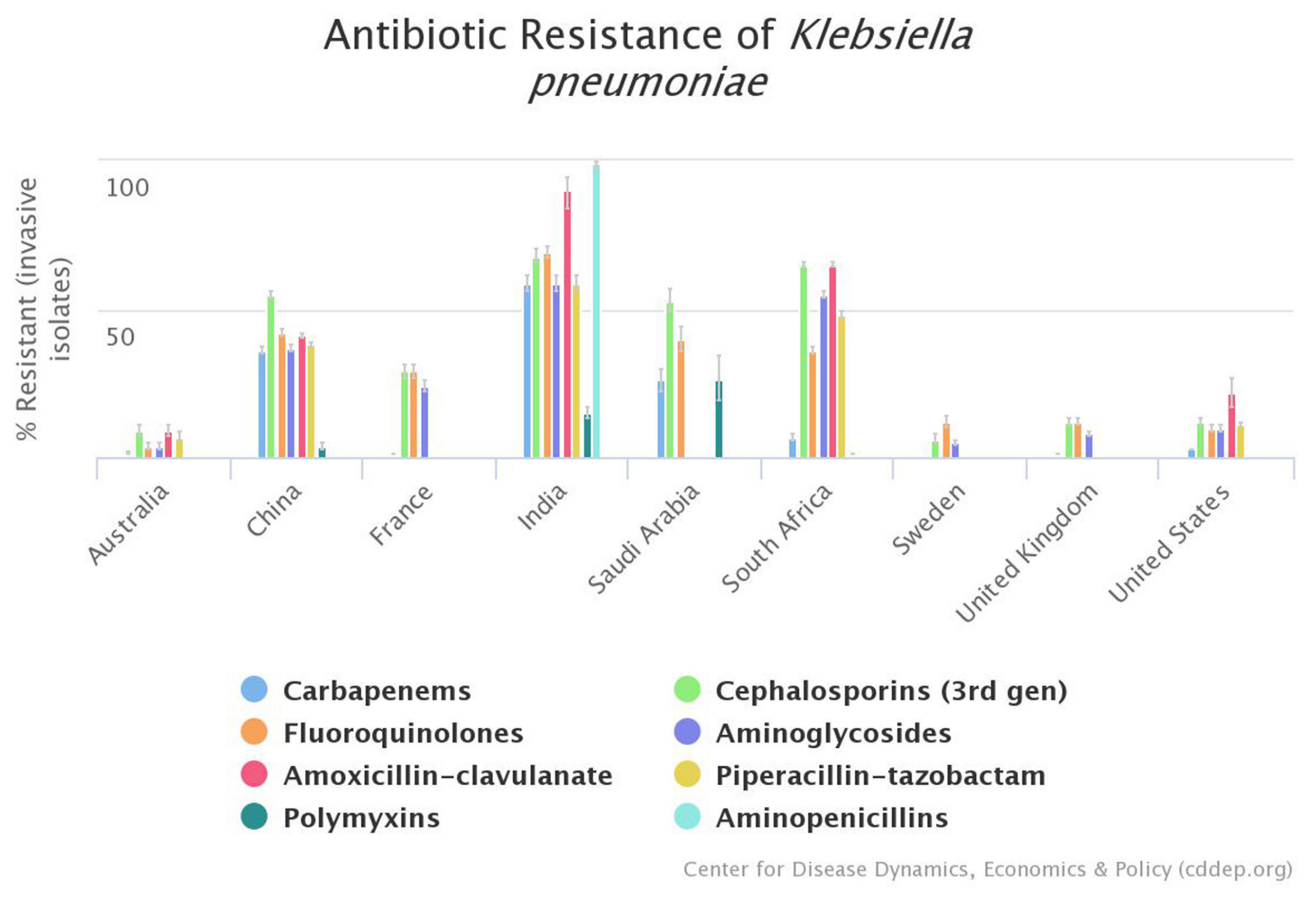
Graphical representation of existing disparity between high-income countries (HICs) and lower-middle-income countries (LMICs) regarding antimicrobial resistance profile of *K. pneumoniae*; according to Center for Disease Dynamics, Economics, & Policy (CDDEP) data (37).

The indiscriminate use of antimicrobials has inadvertently lead to the emergence of resistance to different drugs (32, 36). The stark difference between antibiotic resistance rates of *K. pneumoniae* between HICs and LMICs is evident in Figure 1.

*Klebsiella pneumoniae* is notorious for its ability to acquire antibiotic resistance determinants and it belongs to the ‘critical’ category in the WHO global priority pathogen list (38). It is one of the ESKAPE pathogens [*Enterococcus faecium, Staphylococcus aureus, K. pneumoniae, Acinetobacter baumannii, Pseudomonas aeruginosa, Enterobacter spp*.] which are mostly responsible for the spread of antibiotic resistance in hospital-acquired infections (36). The presence of *Klebsiella* in both the environment and the human body allows them to acquire a large variety of antibiotic resistance determinants. *K. pneumoniae* has a repertoire of around 400 antibiotic resistance genes which is almost double that of other pathogens (24). As the soil and the gut are both hot-spots for the inter-genus transfer of antibiotic resistance, *K. pneumoniae* has a selective advantage as it dwells in both these niches (24). Most of the antibiotic resistance determinants either appear first in *K. pneumoniae* or they are quickly acquired by this organism. *K. pneumoniae* also show higher variability in the G+C contents of its genomes than its other counterparts, indicating that it acquires external DNA from varied sources (24).

As with other Enterobacteriaceae, the majority of antimicrobial resistance (AMR) determinants are plasmid-mediated in *K. pneumoniae* (24, 36). Most pathogenic *K. pneumoniae* carry three or more AMR plasmids and the stability of the plasmids are relatively more in this organism compared to *E. coli* (24). The segment of the plasmid responsible for replication control to maintain a specific plasmid copy number is called a replicon (32). PCR-based replicon typing method (39) and advances in genomics have helped in recognizing plasmid types of *K. pneumoniae*. Various replicons have been found in *K. pneumoniae*, either alone or in combinations, which are IncFIIK, IncN1, IncX3, IncA/C, IncR, IncHI1-FIA, IncHIB-FIB, IncHI2, IncL (32). One of the replicons found frequently in *K. pneumoniae*, IncFIIK replicon, is present in multi-replicon plasmids which also possess IncFIB replicons (32). IncFIIK plasmids have a narrow host range and are rarely found outside this genus. Whereas, other replicons, IncR, IncA/C, IncX3, IncHI1 are of broad host range and thus act as a shuttle for inter-genus horizontal gene transfer (32). Apart from the transfer of genes via plasmids, mobile genetic elements such as insertion elements (e.g., IS*26*), transposons (e.g., Tn*4401a)*, and integrons (e.g., Integron1), present in the plasmids, mediate mobilization of the resistance genes (often gene cassettes) between different plasmids or between chromosome and plasmids (32).

## The Crkp Menace

In the 1980s, extended-spectrum β-lactamases (ESBLs) producing *K. pneumoniae* emerged and spread throughout the world (22, 40). This led to the use of the carbapenems (meropenem, imipenem, ertapenem), a β-lactam antibiotic, which became the antibiotic of choice to treat infections caused by ESBL-positive *K. pneumoniae* (40, 41). Eventually, with use of carbapenems, a new group of enzymes emerged- the carbapenemases, which could hydrolyze most of the β-lactam antibiotics including the carbapenems. The first plasmid-mediated carbapenemase IMP-1 was identified in *K. pneumoniae* in 1991 (42). KPC-1-producing *K. pneumoniae* was reported from the USA in 1996 (43). This carbapenemase was named KPC-1, for *K. pneumoniae* carbapenemase. Since then other carbapenemases have also emerged and CRKP has rapidly spread worldwide (23, 44).

Carbapenem resistance in *K. pneumoniae* or other bacteria occurs by two main mechanisms: (i) production of carbapenemases (45), and (ii) porin loss (OmpK35 and OmpK36) combined with the presence of AmpC cephalosporinases or ESBLs such as CTX-M-15 and/or overexpression of efflux pumps (21, 46, 47).

As carbapenemases are primarily mediated via mobile genetic elements contributing to the spread of carbapenem resistance, a more detailed discussion of these enzymes are done here. Carbapenemases belong to the molecular class A (e.g., KPC, GES, IMI), class B (e.g., IMP, VIM, NDM), and class D (e.g., OXA-48-like) of β-lactamases according to the Ambler classification. Molecular class A and D enzymes are called serine carbapenemases as they contain serine molecule in their active site and molecular class B enzymes are called metallo-β-lactamase (MBLs) as they contain two Zn^2+^ ions in their active site. MBLs cannot hydrolyze the monobactam aztreonam. Class A serine carbapenemases are predominantly inhibited by tazobactam. Class B MBLs are inhibited by EDTA, dipicholinic acid, or 1,10-o-phenanthroline *in vitro*. Class D carbapenemases (e.g., OXA-48-like) which hydrolyze oxacillin, cloxacillin, and carbenicillin, are inhibited *in vitro* by NaCl (46). As different carbapenemases have emerged over time, many have several enzymatic variants (such as KPC-2, KPC-3, NDM-5, NDM-7, OXA-48, OXA-232, IMP-4, IMP-8, etc.) with higher catalytic efficiency, stability, or better metal ion binding capacity (47). All such carbapenemases are harbored on plasmids which have shown both intra- and inter-species transmission.

KPC is a plasmid-mediated molecular class A serine carbapenemase. *bla*_KPC_ gene is found within a unique transposon Tn*4401* variant which has led to the mobilization of the gene to different types of plasmids and through the plasmids to other organisms (32). This carbapenemase has spread vastly in Italy, Greece (48), and the USA (49). Currently, there are 75 alleles reported in the NCBI pathogens database (https://www.ncbi.nlm.nih.gov/pathogens/refgene/#blaKPC), of which KPC-2 and KPC-3 are prevalent. Association of the KPC-2 and KPC-3 with epidemic clone ST258 is the pivotal factor for the spread of these genes.

New Delhi metallo-β-lactamase (NDM-1) is the most widely disseminated class B metallo-β-lactamase. It was first reported in a *K. pneumoniae* and *E. coli* in 2009 and was recovered from a Swedish patient returning from India (50). Although NDM-1 was the most prevalent variant to date, variants such as NDM-4, NDM-5, NDM-7 which are more stable in the zinc-deprived condition due to an M154L mutation have rapidly emerged (51, 52). Currently, there are 31 alleles of *bla*_NDM_ reported in the NCBI pathogens database (https://www.ncbi.nlm.nih.gov/pathogens/refgene/#blaNDM). *bla*_NDM_ is found across various sequence types of *K. pneumoniae*, no association with any particular ST was reported (53). Co-existence of many other antibiotic resistance determinants like *armA, rmtB* (aminoglycoside resistance), *qnrB, qnrS, aac(6*′*)-Ib-cr* (plasmid-mediated fluoroquinolone resistance), and *bla*_CTX−M−15_ (ESBL) (21, 28, 54) is often noticed in these strains. *bla*_NDM−1_ is present in varied broad host plasmids (e.g., IncX3, IncA/C, IncHIB-M/FIB-M) (32). The *bla*_NDM_ gene is almost always found bracketed by a truncated or entire IS*Aba125* element (upstream) and a *ble*_MBL_ gene (downstream) (55).

Molecular class D carbapenemases such as OXA-48 is also a very potent transferable carbapenemase emerging in Enterobacteriaceae (45). It was first reported in 2001 from Turkey in a multidrug-resistant (MDR) *K. pneumoniae* isolate which possessed MBLs and lacked outer membrane proteins (53). OXA-48 has now spread to all continents except Antarctica (56). OXA-48 and OXA-48-like carbapenemases (e.g., OXA-181 and OXA-232) cannot hydrolyze extended-spectrum cephalosporins and can selectively hydrolyze carbapenems (imipenem and ertapenem). Although, *bla*_OXA−48_ is associated with diverse STs, epidemic STs such as ST101, ST147, ST15, and ST395 are more common than others. *bla*_OXA−48_ is generally bracketed by IS*1999* in transposon Tn*1999* and *bla*_OXA−181_ is associated with IS*Ecp1* (57). OXA-181 differs from OXA-232 by a single amino acid substitution and both have a similar genetic environment, suggesting that *bla*_OXA−181_ is the probable progenitor of *bla*_OXA−232._ OXA-181 was first reported from Indian hospitals and is endemic to Indian subcontinent. It is now reported from Asia, Africa, Middle East, Europe, North America, and Oceania (56). OXA-232 was first isolated in France from three patients who just returned from India. The *bla*_OXA−232_ gene was carried in a ColE2 plasmid, situated within a Tn*2013* transposon, downstream a IS*Ecp1* element (58). OXA-232 has been majorly associated with ST14, ST15, and ST16. OXA-232 is endemic in India and has now been reported from other parts of Asia, USA, Africa, and Europe (56).

IMP and VIM are two other plasmid-mediated MBLs. IMP (imipenemase) carbapenemase was first reported in the year 1991 from Japan from an *Serratia marcescens* strain (59). Followed by its identification in *K. pneumoniae* strains from Japan and Singapore, IMP-4-positive *K. pneumoniae* was reported from Australia in 2002 and IMP-8-positive *K. pneumoniae* was reported from Taiwan 2001–2002 and later from various other countries. VIM (Verona integron-encoded metallo-β-lactamases) are spread in Southern Europe and also in other countries (23). VIM-1 and VIM-2 were discovered in *P. aeruginosa* and later found in Enterobacteriaceae. IncN plasmid carrying *bla*_VIM−1_ was later reported from *K. pneumoniae* in Greece (60). Both these enzymes are associated with class 1 integrons and various insertion sequences such as IS*26*, IS*6100* which are associated with specific plasmid types (61).

## Drug-Resistant Bug and the Newborn

Various studies have been published regarding the spread of carbapenem-resistant Enterobacteriaceae but the data on neonatal sepsis is infrequent. Here we present the studies related to neonatal sepsis caused by CRKP (Table 1). We have focused on the major carbapenemases such as KPC, NDM, and to some extent OXA-48, IMP, and VIM. The genetic aspects of these carbapenemases are already discussed in the above section.

**Table 1:** Carbapenem-resistant K. pneumoniae causing neonatal septicemia or intestinal colonization.

**Country**	**Study timeline**	**Year of publication**	**Clinical presentation**	**Source(s)**	**Sequence Types (STs)**	**Carbapenemases identified**	**Other resistance genes identified**	**Plasmid type(s) and integrons**	**References**
India	NA	2011	Sepsis	Endotracheal aspirate and Blood	ND*^a^*	NDM-1	*bla*_CTX−M−15, TEM−1, OXA−1, SHV−1_	NA*^b^*	(62)
Colombia	Aug 2011–Jan 2012	2013	Hypoxic-ischemic encephalopathy, respiratory distress syndrome, necrotizing enterocolitis, and sepsis	Blood	ST1043	NDM-1	*qnrA* and *bla*_SHV._	IncA/C, *IntI*1	(63)
India	2007–2011	2014	Septicemia	Blood	ND	NDM-1	*bla*_CTX−M−15, TEM−1, OXA−1, CMY, SHV−1_, *armA, rmtB, rmtC, aac(6')-Ib*, and *aac(6')-Ib-cr*	IncN, IncHIB-M/IncFIB-M, IncFIIK, IncR	(21, 55)
India	2012	2014	Sepsis	Blood	ND	NDM-1	*bla*_CTX−M−15_	ND	(64)
Nepal	Aug 2011–June 2012	2014	Suspected sepsis	Blood or Cerebro-spinal fluid.	ST15	NDM-1	*bla*_CTX−M−15, SHV−28, TEM−1, OXA−1_, *qnrB1, aac(6')-Ib*, and *aac(6')-Ib-cr*,	Multireplicon plasmid IncHI1B/IncFIB	(65)
Nigeria	Sept 2012–Sept 2016	2014	Sepsis	Blood.	ST476	NDM-5	*bla*_CTX−M−15_, *bla*_OXA−1_, *bla*_OKP−B−6_ *bla*_TEM−1_, *aac(6')-Ib-cr, ble*_MBL_, *qnrB1*, and *sul2*.	IncX3	(66)
Turkey	2013	2014	Colonizer	Rectal swab	ND	NDM-1	*bla*_CTX−M−15, CTX−M−3, SHV−1, SHV−27, OXA−1_, and *rmtC*	NA	(67)
China	2012–2013	2015	Neonatal sepsis, neonatal pneumonia, necrotizing enterocolitis, and respiratory distress syndrome	Blood	ND	NDM-1	*qnrS* and *bla*_CTX−M−15, CMY−4, TEM−1, SHV−1._	ND	(68)
China	Apr 2011–Oct 2013	2017	Neonatal pneumonia	Sputum, blood, Umbilical secretion	ST 20, ST54, ST705, and ST290	NDM-1, IMP-4, IMP-8	*bla*_CTX−M−14, CTX−M−15, TEM−1, DHA−1_	ND	(69)
China	June 2016–Aug 2016	2018	Sepsis, respiratory distress syndrome	Blood Sputum	ST234 and ST1412	NDM-1	*qnrB4* and *bla*_CTX−M−14, SHV−148_,	ND	(70)
China	2015	2018	NA	Blood, urine, sputum, aspiration catheter, and radiant warmer	ST11, ST20, and ST888	NDM-1 and KPC-2	*bla*_CTX−M−14, CTX−M−15, TEM−1_	ND	(71)
China	May 2014–Aug 2014	2018	Septicemia, pneumonia	Blood, Sputum, and Urine	ST1419 and ST101	NDM-1	*bla*_SHV−12, CTX−M−15, TEM−1_	ND	(72)
India	Dec 2015–Jan 2017	2018			ND	NDM-1, NDM-4, and NDM-5	*bla*_OXA_, *bla*_CMY_, and *bla*_SHV_.	IncFIA, IncFIC, IncF, IncK, IncFIB, IncY, IncFIIA,	(51)
India	Jan 2012–June 2014	2019	Septicemia	Blood	ND	NDM-1	*qnrB, qnrS, aac(6')-Ib, aac(6')-Ib-cr, bla*_CTX−M−15, TEM−1, OXA−1, SHV−1_, *armA, rmtB*, and *rmtC*.	IncFIIK and IncHIB-M	(28)
India	July 2016–Dec 2017	2019	Septicemia	Blood	ST29, ST347, ST1224, and ST2558	NDM-1	*bla*_CTX−M−15_, *qnrS1, qnrB1, aac(6')-Ib*, and *aac(6')-Ib-cr*.	IncFIIK	(73)
China	June 2010–Sept 2010	2013	Sepsis	NA	ST11	KPC-2	*bla*_CTX−M−14, CTX−M−15, TEM−1, SHV−11, SHV−12_, *rmtB, aac(6')-Ib-cr*, and *qnrS*	ND	(74)
Jordan	Jan 2012–Dec 2015	2018	Sepsis	Blood	ND	KPC	ESBL genes	ND	(17)
Egypt	Feb 2019–Sept 2019	2020	Sepsis	Blood	ND	KPC, VIM, and NDM	*bla*_CTX−M_, *bla*_OXA−1_, qnrS, and *qnrB*.	ND	(75)
India	2013–2016	2020	Sepsis	Blood	ST147	KPC-2	*bla*_CTX−M_, *bla*_TEM,SHV,OXA_, *qnrB, oqxA, oqxB, aac(6')-Ib-cr*, and *aac(6')-Ib*	IncFII	(76)
China	2018–2019	2020	unknown	Sputum, pus, ascites, urine, blood	ST11, ST76, ST4854, ST35, ST34	KPC, NDM-1,IMP-4	NA	NA	(77)
Egypt	Nov 2015–Apr 2016	2020	Late-onset sepsis	Blood	ND	OXA-48 and NDM	ND	ND	(16)
India	Jan 2016	2020	Septicemia	Blood	ST23	OXA-232	*bla*_SHV−190_, *bla*_TEM−1B_, *bla*_CTX−M−15_, *bla*_CMY−4_, *aac(6')-Ib-cr, oqxB, oqxA*, and *qnrB1*	IncColKP3 type	(78)
China	Apr- June 2016	2017	NA	NA	ST15	OXA-232	*bla*_CTX−M−15_,*bla*_SHV−1_	IncColE type	(79)
Spain	2012–2014	2017	Colonizer	NA	ND	OXA-48	ND	ND	(80)
Algeria	Jan 2017–Apr 2017	2019	Colonizer	Rectal swab	ST13, ST45, and ST1878	OXA-48	ND	IncL/M	(81)
India	2013–2016	2021	Septicemia	Blood	ST14	OXA-181, NDM-5	*bla*_CTX−M−15_, *bla*_TEM−1_, *bla*_OXA−1_, *bla*_OXA−9_, *rmtB, aac(6'-Ib), aac(6')-Ib-cr, oqxA, oqxB*	IncColKP3, IncFII, IncR	(82)
Italy	Apr 2015–Mar 2016	2017	Colonizer	Rectal swab	ST104	VIM-1	*bla*_SHV12_, *ant(3″), aph(3″), aacA4, qnrA1, sul1*, and *dfrA14*.	IncA/C1	(83)
China	Mar 2010–Feb 2011	2014	Respiratory distress syndrome	Trachea cannula	ND	IMP-38	ND	ND	(84)

a *ND, Not determined*.

b *NA, Not available*.

Over the last few years, KPC-producing *K. pneumoniae* have been reported in neonates from various countries such as Egypt (75), Jordan (17), China (71, 74, 77), and India (76). *bla*_KPC−2_-positive *K. pneumoniae* caused infections in China and India. The sequence types of the corresponding strains from China and India were however different: strains from China belonged to ST11 whereas, strains from India belonged to ST147 (76). *bla*_KPC_ was harbored in large plasmids along with other resistance determinants such as *bla*_CTX−M−15, TEM, SHV, OXA−1_, *rmtB, aac(6*′*)-Ib-cr, qnrB*, and *qnrS*. The genetic environment of *bla*_KPC−2_ in ST147 strains from India corroborated with the genetic environment of the other *bla*_KPC−2_-possessing strains recovered from adults, where *bla*_KPC−2_ was associated with IS elements IS*Kpn6* and IS*Kpn7*, plasmid type IncFII, and transposon Tn *4401*. All neonates in this study had an overlapping stay in the hospital so a chance of transmission from one neonate to another was predicted (76). In one of the above studies from China, three of the four infants died due to the infection caused by KPC-producing *K. pneumoniae*. Only one neonate responded to the therapy of amikacin in combination with imipenem (74). The other study from China mentioned the isolation of KPC-producing CRKP not only from blood but also from sputum, urine, aspiration catheter, and hospital environment, indicating that the hospital environment can harbor CRKP strains which may cause disease later (71). The study from Jordan interestingly showed that infection due to KPC-producing *K. pneumoniae* and *Acinetobacter spp*. led to higher mortality and previous exposure to carbapenems and vancomycin significantly increase this risk (17). Further, the study from Egypt showed that neonatal mortality was inversely related to gestational age and birth-weight. The same study also showed that neonates who eventually succumbed to the infection had a significant reduction in platelet count and hemoglobin levels (75).

Shortly after the report of *bla*_NDM−1_ in an adult patient in 2009, *bla*_NDM−1−_possessing *K. pneumoniae* causing neonatal septicemia was identified in 2011 from India (62). Three retrospective studies from the same unit showed that carbapenem-resistant *K. pneumoniae* not only persisted in the unit but gradually became the most predominant carbapenem-resistant organism causing septicemia (21, 28, 55). One of these studies also reported the *in vivo* interspecies plasmid transfer event of *bla*_NDM−1_ in a neonate from whom *Enterobacter cloacae* was isolated initially and *E. coli* later. The study showed that the *bla*_NDM−1_ plasmid in both the species was identical indicating its possible transmission between *Enterobacter cloacae* and *E. coli* (55). The study of Datta et al. reported that male sex, low birth weight, birth at extramural centers were significantly associated with sepsis caused by NDM-1-positive isolates. However, sepsis caused due to these isolates did not result in a higher mortality rate (21). NDM-positive *K. pneumoniae*-mediated neonatal infections are now reported from India (51, 85), Nepal (65), China (68–70, 72, 77), Nigeria (66), Colombia (63), and Turkey (67). Although *bla*_NDM−1_ was the most prevalent allele to date, alleles such as *bla*_NDM−4_ and *bla*_NDM−5_ are slowly emerging in *K. pneumoniae* causing neonatal infections (51, 52). It was observed that isolates harboring *bla*_NDM−1_ are not associated with any particular sequence type and these strains belonged to varied STs (ST15, ST17, ST20, ST29, ST76, ST101, ST234, ST347, ST433, ST476, ST888, ST1043, ST1412, ST1419, ST1224, ST2558, ST4854) (Table 1). These isolates also possessed several antibiotic resistance determinants such as *armA, rmtB* (aminoglycoside resistance), *qnrB, qnrS, aac(6*′*)-Ib-cr* (plasmid-mediated fluoroquinolone resistance), *bla*_CTX−M−15_ (ESBL) (21, 28, 86) along with *bla*_NDM−1_ gene in different plasmid types such as IncFIIK, IncHIB-M, IncFII, IncFIA, IncFIB, IncF, IncA/C, IncL/M, IncA/C, IncX3, etc. (28, 51).

Isolates harboring *bla*_NDM−1_ have also caused outbreaks in several healthcare settings. During August 2011–Jan 2012, an outbreak occurred due to *K. pneumoniae* ST1043 in a neonatal unit in Colombia infecting six neonates. As the neonates had no contact with people from countries that reported NDM-1-producing bacteria, the authors proposed that autochthonous clones were acquiring the *bla*_NDM_ gene (63). Another outbreak around the same time was reported from Nepal by a *bla*_NDM−1_-positive *K. pneumoniae* ST15. This outbreak caused high mortality among the neonates. Apart from the outbreak cluster, three smaller genetically close clusters were also identified in this study (65). An outbreak of NDM-5-producing *K. quasipneumoniae* was reported from Nigeria in April 2016. The outbreak occurred when the neonatal ward was overcrowded and less critical neonates often shared cots. *bla*_NDM−5_ gene was carried on an IncX3 plasmid (66). Five separate studies from different parts of China (Nanjing, Wuhan, Hunan, Jiangsu, Shandong) reported outbreaks of *bla*_NDM−1_-possessing *K. pneumoniae* in neonatal units. Strains were isolated from blood or sputum or umbilical secretions of neonates and belonged to different sequence types (68–70, 72, 77). In another study from China, five CRKP isolated from neonates (blood, urine, and catheter tips) possessed *bla*_NDM−1_ and belonged to ST20 (*n* = 4) and ST888 (*n* = 1), which were susceptible to gentamicin, amikacin, aztreonam, ciprofloxacin, and levofloxacin. NDM-1-producing ST20 strains (*n* = 2) were also isolated from the hospital environment (71). A systematic review from China on carbapenem-resistant Enterobacteriaceae reviewed seventeen studies of neonatal infections among which seven studies were of NDM-1-producing *K. pneumoniae* (87).

OXA-48-positive CRKP causing neonatal infection or intestinal colonization was reported from Algeria, Spain, and Egypt (75, 80, 81). In the study from Algeria, *bla*_OXA−48_-carrying *K. pneumoniae* of two different STs (ST13 and ST1878) were found colonizing the gut of the neonates in two maternity wards. Carriage of carbapenem-resistant strains was significantly related to the low-birth-weight of the neonates (81). In the study from Egypt, *K. pneumoniae* was the predominant organism causing LOS but not EOS. Eventually, the mortality was significantly higher in neonates suffering from LOS (16). OXA-232, another OXA-48-like enzyme, was reported from a hypermucoviscous *K. pneumoniae* causing septicemia from India. The gene was carried in a ColKP3 type plasmid (73). Another clonal outbreak of OXA-232-producing *K. pneumoniae* ST15 was reported from a NICU in Shanghai, China (79).

The other carbapenemases such as IMP or VIM have not been reported frequently in neonatal infections. IMP-38-positive CRKP causing respiratory distress syndrome in neonates was reported from China. The nine IMP-38-positive strains were clonal and were isolated from the trachea cannula of neonates. *bla*_IMP−38_ was a novel allele and differed by a single mutation from IMP-4 which was found in the other wards of the same hospital (84). In another recent study from China, fourteen *bla*_IMP−38_-possessing *K. pneumoniae* ST307 were recovered from neonates suffering from sepsis (88). Another variant, IMP-4 has also been reported from China causing neonatal infections (69, 77, 87). VIM-positive CRKP was recovered from the neonates in the USA, Italy, and Egypt (75, 83, 89). In the study from Italy, VIM-1, associated with an IncA/C plasmid, was primarily found in *K. pneumoniae* ST104 recovered from the rectal swabs of neonates admitted in a NICU from 2015 to 2016. The strains were susceptible to fluoroquinolones, amikacin, and colistin (83).

It is noteworthy that earlier studies reported the presence of a single carbapenemase in *K. pneumoniae* but in recent years reports of co-occurrence of multiple carbapenemases are emerging (16, 75, 82). In a recent study from India OXA-181/OXA-232 was concomitantly present with NDM-5 in *K. pneumoniae* causing neonatal septicemia (82). A study from Egypt also reported (previously mentioned) presence of *bla*_NDM_ and *bla*_VIM_ in *K. pneumoniae* causing LOS. *bla*_KPC_ was also present in 96% of these strains (75). Another (previously mentioned) study from Egypt reported the presence of *bla*_OXA−48_ in 61% strains and co-occurrence of *bla*_NDM_ and *bla*_OXA−48_ in 52% strains (16). The presence of multiple carbapenemases and other resistance genes pose additional limitations to the treatment protocols.

## Hypervirulent *K. Pneumoniae* (hvKP)—Another Dimension to the Problem

### hvKP and cKP: We Beg to Differ

Recently, *K. pneumoniae* has gained a revised and serious global attention due to the emergence of the hypervirulent pathotype. Over the last few decades, the majority of hospital-acquired infections reported globally were due to the classical *K. pneumoniae* (cKP). However, since the mid-1980s, the emergence of hypervirulent *K. pneumoniae* (hvKP) in the clinical context poses a far greater challenge to the clinicians (90). Although, both cKP and hvKP pathotypes have their own global importance, the incidence of infections due to hvKP has been reported at an escalating rate over the last three decades (91). Unlike cKP, hvKP pathotypes are more virulent and have the potential to cause several community-acquired invasive, life-threatening, and unusual infections, such as pyogenic liver abscess, lung abscess, meningitis, endophthalmitis, brain abscess, and necrotizing fasciitis in otherwise healthy adults (92). Initially, hvKP infections were reported primarily from Taiwan and South-East Asia, but several sporadic reports of hvKP have now been observed in other Asian, European, and American countries (25, 91, 93–98). Although hvKP cause community-acquired diseases, some current reports argued that the infiltration of these notorious strains is increasing in the healthcare settings also (99–104). Infections due to hvKP are found to be more complicated due to their ability to metastatically disseminate to other organs or systems and subsequently cause multiple sites of infection (90). This type of dissemination is common for some selected Gram-positive pathogens, such as *S. aureus*, but it is unusual for an enteric Gram-negative bacillus to involve multiple sites of infection (105). In addition, unlike cKP, hvKP pathotypes commonly possess a hypermucoviscous phenotype, produce a robust capsule, synthesize several iron-scavenging siderophore molecules, especially salmochelin and aerobactin, and harbor several chromosomal and large virulence plasmid-encoded factors (91, 106). Due to their enhanced virulence, these *K. pneumoniae* are considered hypervirulent. An overview of the differences between cKP and hvKP strains is depicted in Table 2.

**Table 2:** Characteristic features of classical *K. pneumoniae* (cKP) and hypervirulent *K. pneumoniae* (hvKP) strains.

**Parameter**	**Characteristic(s) for pathotype**	
	**cKP**	**hvKP**
Primary site of acquisition	Nosocomial	Community acquired*^a^*
Population(s) at risk	Immunocompromised individuals	Often otherwise healthy individuals
Liver abscess	Usually do not occur*^b^*	Often occur in the absence of biliary disease
Ability of metastatic spread	None	Frequent
Number of sites of infection	Usually single	Often multiple
Unusual infections	None	Often encountered
Geographical distribution	Worldwide	Mostly Asia-Pacific Rim
Capsule type(s)	K1-K78	Mostly hypercapsule K1 or K2*^c^*
Siderophores	Mostly enterobactin and yersiniabactin	All four siderophores but specifically salmochelin and aerobactin
*magA* and c-*rmpA*	Usually do not occur	Frequently occur
Virulence plasmid-encoded factors (p-*rmpA, rmpA2, iroBCDN*, and *iucABCDiutA*)	Usually do not occur*^d^*	Predominantly occur

a *Recently an escalating number of hvKP infections are emerging in the healthcare settings*.

b *cKP strains can also cause hepatic abscess but unlike hvKP, cKP mediated liver abscess occur in the presence of biliary disease*.

c *Apart from the frequently encountered K1 or K2 serotype, several other capsular type, such as K5, K20, K54, and K57 have also been detected in hvKp strains*.

d *During the course of evolution, cKP strains are also increasingly reported to acquire hvKP virulence plasmid*.

### hvKP and cKP: Molecular Markers

Recently, the advancement of whole-genome sequencing (WGS) in the clinical context has identified a set of important and unique hypervirulent biomarkers which can accurately differentiate hvKP from cKP, including several capsular serotypes (K1, K2, K5, K20, K54, and K57), mucoviscosity-associated gene A (*magA*), regulator of mucoid phenotype A (*rmpA*) genes, biosynthetic genes responsible for the production of siderophore salmochelin (*iro* cluster), aerobactin producing biosynthetic genes (*iuc* cluster), virulence plasmid-encoded gene with unknown function (*peg-344*), and the virulence plasmid-encoded heavy metal resistance genes for tellurite (*ter* cluster) and silver (*silS*) (78, 91, 106). Due to presence of these factors, hvKP strains are more resistant to macrophage- or neutrophil-mediated phagocytosis & the complement-mediated bactericidal activity of human serum, they exhibit enormous biofilm-forming capability, and also display enhanced lethality in the *in vivo* mouse model compared to cKP (107). In the following section, we discuss briefly the several hvKP-specific biomarkers detected till date which are responsible for the increased virulence and severe clinical expression.

#### (a) Capsular Serotypes

Capsule is the most established virulence factor of *K. pneumoniae*. Both cKP and hvKP possess chromosomally-encoded capsular polysaccharide (K antigens) genes, known as the *cps* cluster (108). Till date, around 78 capsular serotypes (K1–K78) have been reported in *K. pneumoniae* (33). However, majority of reports have shown the strong association of K1 and/or K2 serotypes with hvKP strains (109, 110). Recent studies, especially from China, reported the occurrence of K2 serotypes in at least 70% and 42% of hvKP strains (93, 111) and K1 in 24% (93). Why are the increased incidences of virulence associated typically with K1 and K2 serotypes? There are several explanations for this. One study suggested that strains with K1 and/or K2 serotypes have better survival probably because of the induction of slightly greater amount of reactive oxygen species released by neutrophils than other serotypes (112). In addition, several studies have argued that the strains of the K1 and K2 serotypes are significantly more resistant to phagocytosis and subsequently intracellular killing by macrophages and neutrophils than other serotypes (113, 114). Moreover, others have suggested reduced uptake of K1 and K2 serotypes by the innate immune cells probably due to the presence of a significant amount of sialic acid on their surfaces, which may mimic the host cell and allow them to easily evade the immune response (115). Apart from the K1 and K2 serotypes, recent studies revealed the occurrence of other serotypes, such as K5, K20, K54, and K57 in hvKP strains causing various invasive infections (91). However, significant reports of immune evasion are still scarce for these capsular types.

#### (b) Major Players in Hypercapsule Production: rmpA and magA

The capsule is found in both cKP and hvKP, but the hvKP strains produce an increased amount of capsular polysaccharide compared to that of cKP. This robust capsule synthesis in hvKP strains is chiefly mediated by the *rmpA* and *magA* genes. In hvKP strains, a total of three *rmpA* genes are generally found, of which two are large virulence plasmid-encoded (p-*rmpA* and p-*rmpA2*) and one is chromosomally-encoded (c-*rmpA*) (116). Several reports suggested that the *rmpA* genes along with the regulation of capsule synthesis A and B genes (*rcsAB*) can induce the transcription of entire *cps* operon, resulting in hypercapsule production (106). In separate studies, Hsu et al. and Li et al. revealed that about 55–100% hvKP strains express at least one copy of *rmpA* or *rmpA2* (116, 117). In the absence of *rmpA* or *rmpA2*, hypercapsule biosynthesis can be triggered alone by the chromosomally-encoded *magA* gene which was isolated from hypermucoviscous liver abscess-causing *K. pneumoniae* (118, 119). Subsequent bioinformatics and genetic experiments determined that *magA* is a K1-specific factor and encodes a polymerase gene termed *wzy* in the *cps* operon (118, 120, 121).

#### (c) The Iron Scavengers: Aerobactin and Salmochelin

Iron, a crucial and limiting metal, essential for bacterial growth and plays a pivotal role in the progression of bacterial infection, especially in the case of *K. pneumoniae*. However, this essential metal is not readily available in the host during the infection because of the non-specific immune response exhibited by the host where the host efficiently sequesters this metal ion (Fe^3+^) with several iron-binding molecules, such as transferrin and lactoferrin, eventually restricting the growth of many opportunistic pathogens (122). Therefore, to acquire iron from such an iron-poor environment, *K. pneumoniae* secrete several small proteins with high iron-scavenging ability, called siderophores (123). Molecular epidemiological studies have shown that hvKP strains produce all the siderophores (enterobactin, yersiniabactin, salmochelin, and aerobactin) compared to cKP which produce only enterobactin and yersiniabactin (124). Apart from producing all siderophores, hvKP strains are also capable of synthesizing quantitatively more siderophores than cKP (125, 126). Among the four siderophores, the activity of enterobactin and yersiniabactin is greatly hindered by host molecule lipocalin-2 and transferrin, respectively (127, 128). Therefore, *K. pneumoniae* with only enterobactin and/or yersiniabactin are unlikely to cause systemic infection in immunocompetent individuals (128). In contrast, the functionality of both salmochelin and aerobactin cannot be inhibited by these host proteins. Several studies have shown that salmochelin is more prevalent in hvKP strains, sometimes more than 90% of pyogenic liver abscess-causing hvKP strains possess this scavenger (128, 129). Aerobactin, a citrate-hydroxamate siderophore, is also rarely detected in cKP (124). Studies revealed its presence in 93–100% of hvKP strains (130). Immense genome-based analysis in the molecular epidemiologic studies confirmed that in most cases *iro* gene cluster for salmochelin and *iuc* gene cluster for aerobactin specifically reside on the large virulence plasmid of hvKP (131).

#### (d) Plasmids That Matter: pLVPK

**Figure 2:**
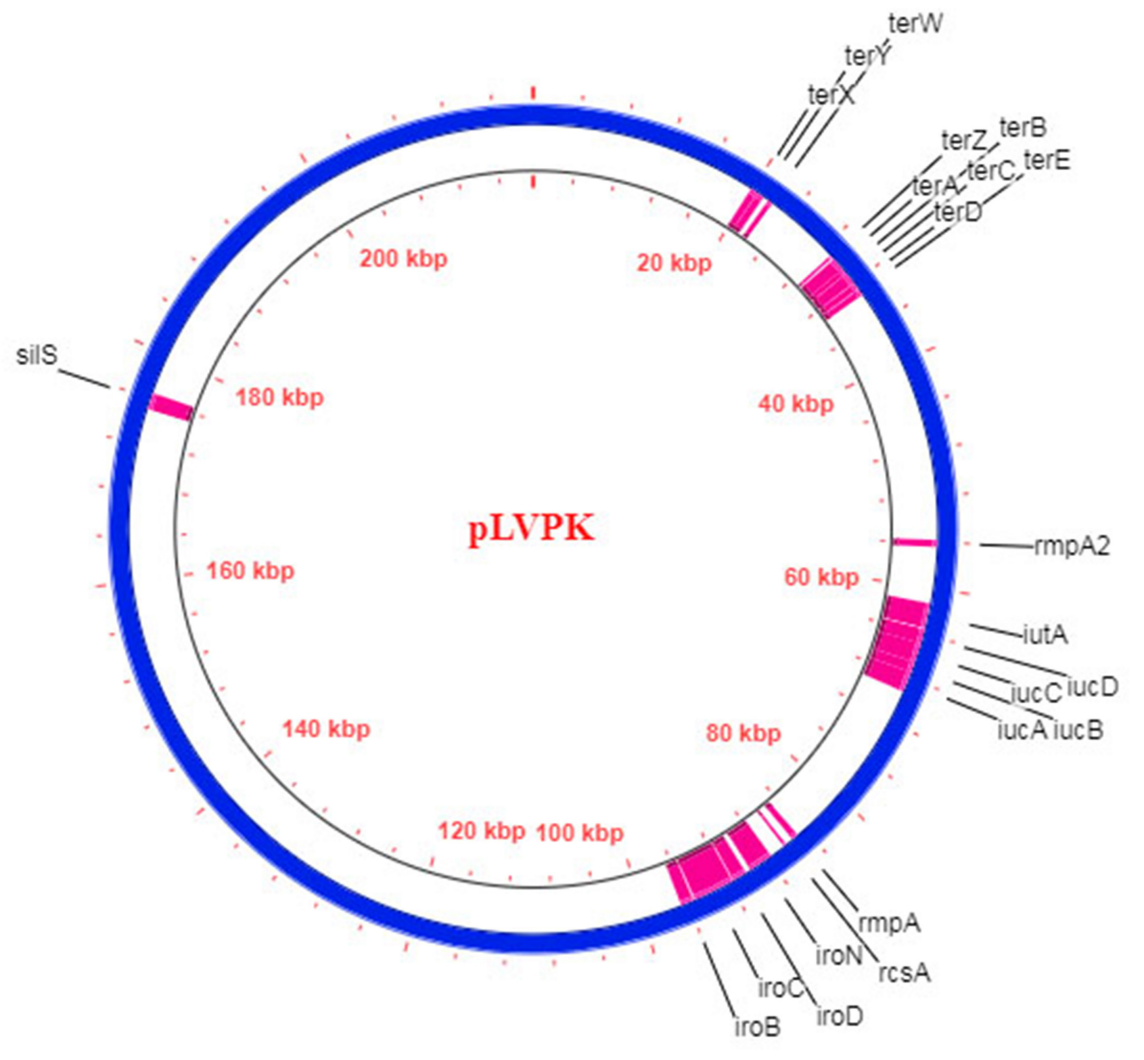
Schematic diagram of the hvKP virulence plasmid pLVPK (Blue circle; 219,385 bp) (35). The respective CDS of the hypervirulent biomarkers are demarcated in pink.

WGS analysis of initially identified hvKP strains revealed the presence of the unique ~220 kb large virulence plasmids pK2044 (224,152bp) and pLVPK (219,385bp) (34, 35). All the best described genetic markers which confer the hypervirulent phenotype are located on these plasmids, including complete biosynthetic gene cluster of aerobactin (*iucABCDiutA*) and salmochelin (*iroBCDN*), regulators of hypercapsule production (*rmpA, rmpA2*, and *rcsA*), resistance genes for tellurite (*terZABCDE* and *terWXY*) and silver (*silS*) (Figure 2). Recently, bioinformatics studies revealed that all the hvKP strains possessed either pK2044-like or pLVPK-like plasmid. Struve et al. showed that all 30 studied community-acquired liver abscess-causing hvKP strains harbored pLVPK-like plasmids (131). Similarly, another study revealed the presence of pK2044-like plasmids in 94 hvKP strains (132). The lateral gene transfer in bacteria is largely mediated by plasmids and as the hypervirulent biomarkers mostly reside in the non-conjugative virulence plasmids, it is quite likely that hvKP strains acquire drug-resistant plasmids from the cKP strains due to their conjugative nature and this molecular incidence is now increasingly evident in recent studies.

#### (e) hvKP and Sequence Type 23 (ST23): In Search of an Association

Several studies showed that the genes conferring hypervirulence are widely distributed across diverse STs (133) but some selected STs, such as ST23, ST65, and ST86 are found to be predominantly associated with hvKP (134). Recently, core genome multi-locus sequence typing (CG-MLST) and/or WGS revealed that strains of clonal group 23 (CG23) are strongly associated with K1 capsular type causing severe and invasive disease which occur in typical hvKP infection (131). ST23 was frequently associated with hvKP strains especially in the Asia-Pacific Rim. Studies from China and South Korea show that majority of the hvKP strains belonging to ST23 possessed K1 capsular type (134, 135). Recently, a study from India in 2020 also described a case of community-acquired neonatal septicemia caused by a carbapenem-resistant ST23 hvKP strain (78). Although the reasons behind the association of the hvKP and/or K1 capsular type with ST23 are still uncertain, it is hypothesized that the CG23 probably has a discrete genetic infrastructure that confers the hypervirulence. However, in-depth research will be required to fulfill the existing knowledge gaps.

## The Next Generation Super-Bug: Carbapenem-Resistant hvKP (CR-hvKP)

**Figure 3:**
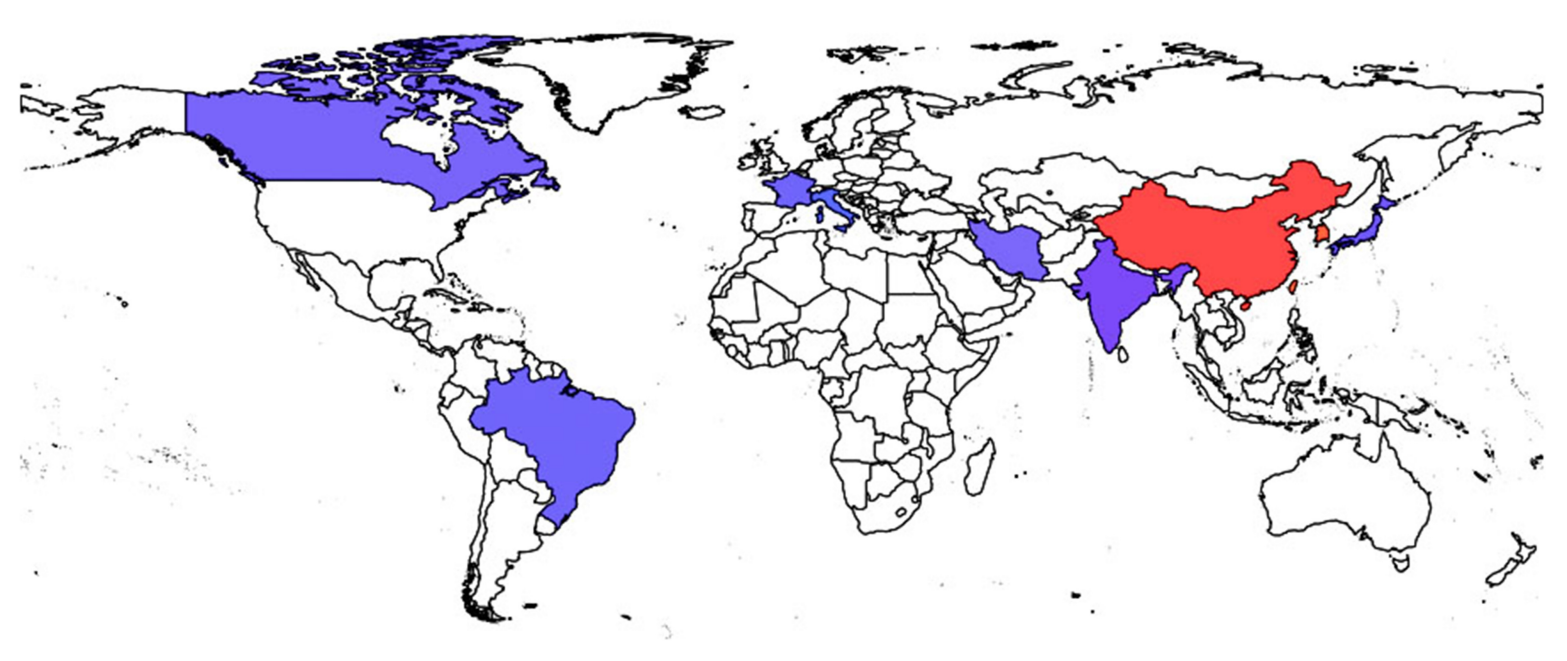
Worldwide spread of CR-hvKP strains. Endemic spread of CR-hvKP strains were reported from the countries indicated in red while the blue colored regions represent the sporadic occurrence of CR-hvKP strains.

It is debatable whether the association of virulence and antibiotic resistance is deleterious or helpful for the microbe (90, 124). However, recent reports on *K. pneumoniae* that are both antimicrobial-resistant and virulent have surely put an end to this debate. *K. pneumoniae* are extremely capable of receiving and incorporating DNA segments from other bacteria, mostly via large plasmids (26). Carbapenem resistance genes, as discussed earlier can easily spread across species via these mobile genetic elements. Their presence in hvKP has challenged the health system and the emerging CR-hvKP pathotypes are now being considered as “the next generation super-bug.” The epidemiological data of some CR-hvKP strains is given in Table 3 and the worldwide distribution of CR-hvKP strains is depicted in Figure 3.

**Table 3:** Epidemiology of some carbapenem-resistant hypervirulent *K. pneumoniae* (CR-hvKP) strains.

**Patient population**	**Country**	**Sequence types (STs)**	**Capsule type(s)**	**Hypervirulent markers**	**Associated carbapenemase**	**Ambler class**	**Year**	**References**
Neonates	India	ST2318	ND*^a^*	*rmpA, rmpA2*, aerobactin, and salmochelin	NA*^b,#^*	NA	2016	(136)
	Russia	ND	ND	*rmpA*	NA*^#^*	NA	2017	(137)
	Russia	ND	ND	*rmpA*, and aerobactin	NA*^#^*	NA	2018	(5)
	India	ST23	K1	*magA, rmpA, rmpA2, iucABCDiutA*, and *iroBCDEN*	*bla*_OXA−232_	Class D	2020	(78)
	Sudan	ND	K2	*magA* and *rmpA*	*bla*_NDM_ and *bla*_OXA−48−like_	Class B and D	2020	(138)
	India	ST5235	ND	*rmpA* and *rmpA2*	*bla*_NDM_ and *bla*_OXA−48−like_	Class B and D	2021	(139)
Adults	China	ST11, ST25, and ST65	K2 and non-typeable	*rmpA, iucABCDiutA*, and *iro*	*bla*_KPC−2_	Class A	2015	(140)
	China	ST23 and ST1797	K1	*magA* and *rmpA*	*bla*_KPC−2_	Class A	2015	(141)
	China	ST11	K1	*magA, rmpA, rmpA2, iro*, and*iucABCDiutA*	*bla*_KPC−2_	Class A	2016	(142)
	China	Unknown	K1	*rmpA* and *iucABCDiutA*	*bla*_KPC−2_	Class A	2017	(143)
	China	ST11, ST268, ST65, ST692, and ST595	K2, K20, and non-typeable	*rmpA* and *iucABCDiutA*	*bla*_KPC−2_	Class A	2017	(144)
	China	ST11	K47	*iucABCDiutA* and *rmpA*	*bla*_KPC−2_	Class A	2017	(145)
	China	ST15	KL112*^c^*	*rmpA2* and *iucABCDiutA*	*bla*_OXA−232_	Class D	2018	(146)
	Iran	ST23	K1	*rmpA, magA*, and aerobactin	*bla*_VIM−2_	Class B	2018	(147)
	China	ST86	K2	*rmpA, iucABCD*, and *iroBCDN*	*bla*_NDM−1_ and *bla*_KPC−2_	Class A and B	2018	(148)
	Canada	ST86	K2	*rmpA, rmpA2, iucABCD*, and *iroBCDN*	*bla*_KPC−2_	Class A	2019	(149)
	Japan	ST23	K1	*rmpA, rmpA2* (with frameshift)*, iucABCD*, and *iroBCDN*	*bla*_IMP−6_	Class B	2019	(150)
	Singapore	ST23, ST65, and ST86	K1 and K2	*rmpA, rmpA2, iuc*, and *iro*	*bla*_KPC−2_	Class A	2019	(151)
	Singapore	ST23, ST65, ST86, ST420, and ST893	K1, K2, and K20	*rmpA, rmpA2, iuc*, and *iro*	*bla*_KPC−2_	Class A	2020	(152)
	France	ST86	K2	*rmpA, rmpA2, iucABCDiutA*, and *iroBCDEN*	*bla*_OXA−48_	Class D	2020	(153)
	China	ST1764	K64	*rmpA, rmpA2*, and *iroN*	*bla*_SIM_	Class B	2020	(154)

a *ND, Not determined*.

b *NA, Not available*.

# *ESBL-producing hvKP strains*.

c *KL means K Locus: associated cps cluster type*.

From the standpoint of the microbe, the convergence of resistance and virulence is beneficial particularly under antibiotic pressure. Exchange of genes can happen in two ways, hvKP strains can acquire the antibiotic-resistant plasmids from the XDR *K. pneumoniae* strains (155) or the XDR cKP strains can acquire hvKP-specific virulence plasmids (145). The first option seems more plausible given the fact that majority of pLVPK-like plasmids are non-conjugative, lacking the transfer (*tra*) genes (131, 134). On the other hand, carbapenem resistance genes are primarily found on conjugative plasmids. However, some recent in-depth investigations on hvKP revealed that due to the immense antibiotic selection pressure and an extraordinary ability of *K. pneumoniae* to acquire highly antibiotic-resistance- and hypervirulence-encoding genetic determinants, new hybrid and/or conjugative plasmids with both carbapenem-resistant and hypervirulent markers have emerged in the *K. pneumoniae* strains as a result of convergent evolution. This molecular phenomenon has eventually led to the emergence of new and perilous variants of CR-hvKP strains (156, 157).

Since 2015, multiple epidemiological studies from China showed the prevalence of KPC-2-producing CR-hvKP strains in the clinical settings causing several infections in otherwise healthy individuals, including UTI, pneumonia, septicemia, bacteremia, abdominal infections, septic arthritis, catheter-associated bacteremia, and ventilator-associated pneumonia (140–142, 144). Majority of the reported *bla*_KPC−2_-harboring CR-hvKP strains from China belonged to ST11. Although ST11 clone of *K. pneumoniae* is an internationally categorized high-risk clone known to harbor *bla*_KPC−2_ as the major carbapenemase gene, these recent studies have confirmed its association with the hypervirulence attributes.

Typical to hvKP, pLVPK-like virulence plasmid pVir-CR-hvKP4 was identified. However, compared to the pLVPK, pVir-CR-hvKP4 had a 41.231 bp deletion. The subsequent plasmid-curing experiment suggested that this modified pLVPK-like plasmid was responsible for enhanced virulence phenotype both *in vitro* and *in vivo* (145). Although majority of the CR-hvKP reports were initially detected with *bla*_KPC−2_, recently the occurrence of other classes of carbapenemases (Class B -MBL-type and Class D—OXA-48-like) have also been detected in hvKP strains. For example, a recent study from China showed the emergence of an ST86 CR-hvKP K2 strain that co-harbored two different classes of clinically important carbapenemase-producing genes *bla*_NDM−1_ and *bla*_KPC−2_. This strain was also responsible for significant mouse lethality (148), refuting the fact that association of resistance and virulence is deleterious for the microbe.

## hvKP/CR-hvKP and the Newborn

Till date, majority of the cases of hvKP and/or CR-hvKP infections have been reported in adults and reports regarding hvKP infection in cases of neonatal sepsis are just a handful (Table 3). When resistance and virulence meet to cause infection in a vulnerable population, the outcome is fatal, as was evident from most neonatal studies (5, 137, 139). Previous studies have concluded that the risk of development of severe forms of neonatal infection can be associated with virulence factors (5). In 2016, a study from India reported the occurrence of an ESBL-producing hvKP ST2318 strain causing neonatal sepsis. The strain was isolated from the blood of a very low-birth-weight neonate suffering from hepatosplenomegaly with severe thrombocytopenia, coagulopathy, and metabolic acidosis. Cefoperazone-sulbactam and amikacin were administered as an empirical treatment which was later changed to meropenem with amikacin after receiving the clinical microbiology report. The neonate was discharged after proper medical supervision. Genome analysis revealed that the strain harbored *rmpA, rmpA2*, and several siderophores, including aerobactin, enterobactin, yersiniabactin, and salmochelin (136). Khaertynov et al. in 2017 reported another fatal case of pyogenic neonatal meningitis caused by an ESBL-producing hvKP. The strain was isolated from the blood and cerebrospinal fluid of a 12-day-old neonate exhibiting several clinical symptoms, such as pallor, loss of appetite, fever, seizures, and a bulging anterior fontanelle. In addition, low Pediatric Glasgow Coma Scale (PGCS) score indicated that the neonate suffered from severe brain injury. Though the newborn was initially treated with ampicillin and amikacin it was changed to meropenem and later cefoperazone due to unsatisfactory clinical outcome and isolation of ESBL-producing *K. pneumoniae* from the infection sites. Apart from antibiotic treatment, IgM-enriched intravenous immunoglobulin and infusion therapy were also given to the neonate but the neonate succumbed to the infection. Laboratory-based characterization revealed that the strain exhibited hypermucoviscous phenotype and also harbored *rmpA* gene (137). The following year another report from Kazan, Russia revealed the occurrence of ESBL-producing neonatal septicemic hvKP strains in the clinical settings with fatal outcome, harboring *rmpA* and aerobactin biosynthetic genes. In this study, two groups of neonates were registered, infected by *K. pneumoniae*. The first group comprised of 10 neonates with culture-positive sepsis and the second group consisted of neonates with UTI. All the 10 septicemic neonates received a comprehensive treatment, including antibiotics, intravenous immunoglobulins, and infusion therapy. However, three neonates died despite the therapy, of which two suffered from purulent meningitis and one from necrotizing enterocolitis. The most grievous forms of sepsis in neonates, were correlated with meningitis (5). The first report of CR-hvKP was in 2020 from our laboratory showing the occurrence of a unique community-acquired neonatal septicemic case caused by a hypermucoviscous CR-hvKP ST23 K1 strain. The neonate was low-birth-weight, delivered at home in a rural area and was given cow milk since birth. During admission, the baby displayed several clinical signs and symptoms of sepsis. As an empirical treatment, piperacillin/tazobactam and netilmicin were given to the neonate. The baby was hospitalized for 22 days before taking a high-risk discharge. The strain displayed all hvKP-associated features, including hypermucoviscous phenotype, *magA, rmpA, rmpA2, rcsAB, iroBCDEN*, and *iucABCDiutA*. In addition, the strain also harbored *bla*_OXA−232_ as the candidate carbapenemase gene. Molecular typing and WGS analysis revealed that the studied strain belonged to an internationally high-risk clone, ST23 and possessed a pLVPK-like virulence plasmid sequence in the studied genome. Moreover, comparative genomic analysis with the other hvKP ST23 reported genomes showed >99.5% of inter-genomic resemblance (78). The virulence determinants such as *magA, rmpA, rmpA2, iroN, iucA*, and *iutA* were not transmitted along with the carbapenem resistance gene, *bla*_OXA−232_, in conjugation experiments. However, the possibility of such transmission is worrisome. The same year another study from Khartoum, Sudan revealed that about 16.7% CR-hvKP strains were recovered from the blood of septicemic neonates and adults. Molecular characterization suggested that among the recovered CR-hvKP strains, eight were positive for *bla*_OXA−48−like_ gene and two for *bla*_NDM_. Moreover, the strains belonged to K2 capsular serotype and also harbored *rmpA* and *magA* (138). In 2021, a study from India showed the occurrence of nine XDR hvKP ST5235 strains causing sepsis in neonates. All the neonates in this study were empirically treated with piperacillin/tazobactam and amikacin followed by imipenem/meropenem and vancomycin. However, 100% neonatal mortality was recorded in this study even after the treatment with polymyxin B. Molecular characterization showed that the strains harbored *rmpA*/*rmpA2* in various combinations. Although all strains showed resistance against carbapenems in phenotypic tests, *bla*_OXA−48−like_ gene and *bla*_NDM_ were detected only in three strains (139).

Clearly, more and more reports of hvKP and CR-hvKP in cases of neonatal sepsis are being published. Clinical laboratories do not evaluate *K. pneumoniae* on the basis of their hypervirulent features and thus such strains are underreported. In neonates, the combined effect of an immature immune system, use of several invasive devices, the involvement of extensively drug-resistant cKP, and the emergence of hvKP/CR-hvKP is worrisome. Comprehensive analysis of cKP and hvKP, their differences, and also the distinguishing biomarkers for hvKP will probably help the clinicians in future to take a more strain-specific therapeutic approach.

## The Possible Acquisition of cKP and hvKP in Neonates

Just like the other members of the Enterobacteriaceae family, *K. pneumoniae* is also found in the normal human microbiota (158). Although the acquisition of *K. pneumoniae* appears to be obligatory for the infection, this scenario is not obvious always (159). The time of pathogenic exposure, bacterial inoculum size, immune status of the individual, and the virulence potential of the causative microorganism collectively manifest the clinical expression of an infection (3). At birth, the neonates do not possess their endogenous microbiota which is naturally acquired through the perinatal transfer of the maternal flora and from environmental sources. Due to the lack of an established microbiota, immaturely developed gut barrier, and the high permeability of the mucosa in the GI tract, neonates get infected easily, particularly low-birth-weight and premature neonates requiring prolonged hospitalization (160). The probable modes of infiltration of *K. pneumoniae* in the neonates are largely accomplished by *in-utero* acquisition, acquisition from maternal flora, and postnatal acquisition from the hospital or community (3).

### (a) *K. pneumoniae* Acquisition From Maternal Flora

*In-utero* infection in neonatal septicemic cases is largely reported to be a result of chorioamnionitis (161). This is an acute inflammation of fetal membranes, probably due to the bacterial infiltration and this clinical condition is often found to be caused by two specific genital mycoplasmas, such as *Ureaplasma parvum* and *Ureaplasma urealyticum* (3). The vaginal microenvironment also provides a suitable ground for the colonization of pathogenic bacteria. Ascending movement of these microorganisms followed by the premature rupture of amniotic membrane results in the contamination of amniotic fluid. The inhalation or swallowing of the infected amniotic fluid by the neonate can subsequently lead to the colonization of pathogenic *K. pneumonia*e in their gut which can lead to intrapartum sepsis (162–164). However, reports regarding *in-utero* infection due to *K. pneumoniae* are extremely scarce. Two exclusive studies confirmed the occurrence of *K. pneumoniae* in intrauterine infection. In the first study in 2005, Sheikh et al. reported a unique case of acute chorioamnionitis and acute villitis due to *K. pneumoniae* infection in a 40-year-old woman at 18 weeks of gestation (165). Torabi et al. also reported a case of severe chorioamnionitis with umbilical cord and chorionic plate vasculitis due to *K. pneumoniae* in a woman who had suffered from fetal expiration at 20 weeks of gestation (166).

Acquisition can also frequently happen during the process of vaginal delivery, the neonates might get infected by the pathogens residing in the birth canal (167). Besides, the neonates can also acquire maternal flora through the contaminated hands, from the skin during the kangaroo care, and/or from the infected breast milk. Recent studies have revealed the incidence of acquisition of ESBL- or carbapenemase-producing *K. pneumoniae* from maternal flora to the neonates. For example, a study from Italy in 2017, reported a case of mother to child transmission of KPC-3-producing CRKP at birth (168). In 2018, Herindrainy et al. reported that the acquisition of about 24% (*n* = 20) of ESBL-producing *K. pneumoniae* detected in the neonates were from the maternal gut flora (169).

### (b) Nosocomial Acquisition of *K. pneumoniae*

Till date, the majority of neonatal septicemic cases from LMICs are reported to be caused by hospital-associated drug-resistant classical *K. pneumoniae* strains which we have already discussed earlier. In several studies, we also have noticed the prevalence of hospital-acquired *K. pneumoniae* and/or CRKP infections in the neonates. The MDR or XDR nature of the *K. pneumoniae* strains offer them an immanent selective advantage by which they can easily persist in the flora of hospitalized patients as well as in the nosocomial environment (170). In a study from India, Das et al. revealed that among the studied bacterial strains, *K. pneumoniae* was detected as the predominant microorganism colonizing the neonatal gut. Neonates with longer stay in the NICU and those with prolonged feeding through an enteral tube were predisposed toward such colonization. Moreover, molecular typing showed that about 50% of the *K. pneumoniae* strains isolated from the blood were genotypically identical with their gut counterparts, reflecting the possible association between gut colonization and neonatal sepsis (162).

### (c) Acquisition of *K. pneumoniae* From the Community

Till date, the majority of community-acquired *K. pneumoniae* have been reported to cause infections in healthy adult individuals. But the reports regarding community-acquired *K. pneumoniae* infections in neonates are very limited. In a study, Waters et al. showed that about 11.6% of *K. pneumoniae* strains caused community-acquired neonatal sepsis in the LMICs (171). In another study, Khatri et al. reported a case of community-acquired pyelonephritis caused by a KPC-2-producing *K. pneumoniae* (172). Recently, in a study we reported a unique case of CR-hvKP, causing sepsis in a neonate who was delivered at home and was given cow milk after birth (also discussed earlier). In that study, we hypothesized that the consumption of cow milk after birth was probably a cause of community-acquired hvKP infection (78). Due to various factors like low female literacy rate, unavailability of clinicians and other healthcare workers, the inadequate number of hospitals and/or well-equipped healthcare facilities, improper transport system, and most importantly inequality in the society, some of the neonates in the LMICs are still delivered either at home or at resource-poor primary care facilities where they might acquire infections from the environmental sources.

## Detection of CRKP and hvKP: Seek and You Shall Find

The initial detection of carbapenemase-producing organisms in a lab is carried out by the trusted disk diffusion method which uses a carbapenem disk placed on a lawn of sample organism (173, 174). If there is a zone of inhibition, the diameter is measured and according to the cut-off mark delineated by CLSI (175) or EUCAST (176), the organism is declared resistant, or intermediate, or susceptible. Automated machines such as VITEK® 2 from bioMérieux and Phoenix™ from BD are also frequently used in setups that can afford them. Phenotypic detection methods (though some are still not cost-effective) may be considered in resource-poor settings. A suitable test should be chosen based upon the sensitivity, specificity, turn-around time, and most importantly, cost of the test. Some phenotypic methods suitable for detection of carbapenemase-producing organisms are presented here in brief, exhaustive reading on these methods are available elsewhere (177). For easy detection of KPC and MBLs, combined disk test was developed. This test uses a disk of meropenem and another disk of a meropenem supplemented with an inhibitor (EDTA/ aminophenyl boronic acid/ dipicholinic acid). If the difference between the zone of inhibition of the two disks is >5 mm, a positive result is indicated (178). These tests are now commercially available (179, 180). mCIM uses a simple method and has high sensitivity and specificity. In this test, a meropenem disk is incubated with 1 μl loopful bacterial culture in Tryptic soy broth for 4 h. After that the disk is placed on a plate spread with a susceptible isolate. If the initial sample is carbapenem-producer then the disk is inactivated and zone of inhibition will be absent on the plate. Both these tests yield results after an overnight incubation (181). On the other hand, CarbaNP test uses the property of a carbapenemase-producer to hydrolyze imipenem within 2 h (182). This hydrolysis produces an acid which lowers the pH and triggers phenol red to change its color from red to yellow. There are several variants of this test. These tests detect all carbapenemases but has low sensitivity for OXA-48-like carbapenemases. The modified CarbaNP test uses a different buffer (0.02% cetyltrimethylammonium bromide lysis buffer) of higher (pH 7.8 instead of pH 7.5) and has higher sensitivity than CarbaNP test. For *K. pneumoniae* the modified test is beneficial as mucoid cells tend to give false negative results in the original test. To differentiate between classes of carbapenemase, CarbaNP test II is used which discriminates based on the use of inhibitors tazobactam (for KPC) or EDTA (for MBLs) (177).

Colorimetric methods to detect carbapenemases directly from blood without blood culture are cost-effective and less time consuming (3–4 h). A study evaluated two variations of CarbaNP, CarbaNPT-direct which uses Triton X-100 as enzymatic extractor and Blue-carba which uses bromothymol blue instead of phenol red, for detection of carbapenemases directly from blood samples (183). The study reported high sensitivity for MBLs and KPC by colorimetric assays but less sensitivity for detection of GES and OXA-48 carbapenemases. A new method with increased sensitivity is the CarbaLux test which uses a fluorescent carbapenem substrate and substrate with cloxacillin to detect not only all carbapenems but also carbapenem-hydrolyzing AmpC enzymes from a bacterial culture (184). Another highly sensitive and specific method is lateral flow immunoassay which detects widely spread carbapenemases NDM, KPC, OXA-48-like, IMP, VIM on a single strip within 15 min from a bacterial culture (185, 186). These require minimal infrastructure.

Several phenotypic tests and commercial kits are available for the detection of CRKP strains (179, 180), such tests and/or kits are yet unavailable for the hvKP strains. The clinical microbiology laboratories are thus, not equipped to distinguish cKP from hvKP during routine diagnosis. Since the hvKP strains are often found to harbor hypercapsule, the hypermucoviscosity appearance of the colonies on the agar plate can be determined by a string test in which a viscous string is generated (>5 mm) when a colony is stretched by an inoculation loop (90). However, the strain discrimination based on only the hypermucoviscous appearance of the colonies and/or the positive string test can be misleading because studies have argued that hypermucoviscosity and hypervirulence are two different phenomena (91, 187). It is noteworthy that several *rmpA* and/or *rmpA2*-positive non-hypermucoviscous strains and also *rmpA* and/or *rmpA2*-negative hypermucoviscous strains were detected in causing invasive syndrome (103, 188, 189). hvKP strains produce quantitatively more siderophores than cKP, and the siderophore assay can be an option for detection. However, as of date, the conclusive discrimination of hvKP is carried out by genotypic methods, such as via specifically amplifying several hvKP-associated genes (*magA, rmpA, rmpA2, iroB, iroN, iucA*, and *iutA*) using polymerase chain reaction (PCR). These hypervirulent biomarkers hold the promise of a sensitive and specific diagnostic test in future. Since, the methodology is largely genotype-based, it is restricted to the research laboratories and may not be readily available in the healthcare settings of LMICs. Research is on-going for the development of a cost-effective commercial test which will not only help the clinical laboratories but can also be used in surveillance and research studies (189). In some resource-poor settings where the basic blood culture facilities are still unavailable or not properly standardized, the detection of either CRKP or hvKP is far from sight. Moreover, the major hindrance in detecting hvKP is probably the lack of awareness of such strains and the need to detect them.

## Treatment: Is There a Silver Lining?

Treatment of neonatal sepsis is a great challenge, the signs of the disease are non-specific, the pathogens are numerous and drug-resistant, the diagnosis has limitations and the patient is vulnerable. Antibiotics remain the primary treatment with supportive respiratory and circulatory treatments along with treatment for metabolic derangements. In contrast to the well-established supportive treatments, the protocols for antimicrobial treatments are often found to be inadequate and region-specific. The choice of antibiotics largely depends on the etiology of the prevailing pathogens, their antimicrobial susceptibility profiles, the age of onset of sepsis, and the microbiology laboratory support available (3). The protocol is often specific for a given unit and greatly varies between and within countries. In this review, we discuss the limitations of the currently available antimicrobial therapy particularly in the LMICs in the current situation of drug resistance.

## Empirical Antibiotic Therapy: Are the Guidelines Still Adequate for Neonates?

In general, the treatment of neonatal sepsis can be broadly divided into suspected or empirical therapy and known or definitive therapy. In clinical microbiology, it is always recommended to obtain the blood cultures for ascertaining bacterial sepsis before the initiation of antimicrobial therapy. However, culture results often require at least 48–72 h to be reported. Considering the non-specific clinical manifestations of neonatal sepsis, the initial antimicrobial therapy should not be unnecessarily delayed for the culture report in case of severely ill and high-risk neonates. In most cases the treatment should start within 1 h of decision to treat (190). This initial antibiotic therapy which is implemented before obtaining the blood culture report is defined as the empirical therapy. Empirical antibiotic therapy should be guided by the prevalent spectrum of pathogenic bacterial strains and their resistance profiles commonly detected in the given NICU or in the community settings. Microbiology support is varied and insufficient in resource-poor settings and treatment is largely empirical in such situations.

Antibiotic resistance has limited the therapeutic options in neonates and this problem is compounded in resource-limited heath infrastructure in LMICs. CRKP strains in neonatal units have challenged the healthcare settings, as these strains not only produce broad-spectrum carbapenemases but also harbor a repertoire of other plasmid-mediated resistance determinants that confer resistance to almost all clinically important antimicrobial classes, including third- and/or fourth-generation cephalosporins, cephamycins, aztreonam, aminoglycosides, and fluoroquinolones (67). Under these circumstances, the WHO treatment guidelines for neonates were found to be ineffective for the LMICs.

In response to the antibiotic resistance crisis, recently, the WHO has revised the treatment guidelines and launched a global action plan on antimicrobial resistance. The aim of this global campaign is to reduce the spread of AMR through optimizing the use of antibiotics, and also to reduce the adverse events and overall costs. The WHO Essential Medicine List (EML) Working Group adopted a tool, AWaRe. This tool classifies the antibiotics into three groups: *A*ccess, *Wa*tch, and *Re*serve (191). The Access group generally includes narrow-spectrum antimicrobials against a wide range of commonly encountered susceptible pathogens. The WHO EML enlisted 19 antibiotics recommended as first or second choice of empiric treatment options for clinical infections. These antibiotics are affordable, greatly assured, and are generally available at all times. The Watch group contains broader spectrum antimicrobial classes that have higher resistance potential and includes most of the highest priority agents among critically important antimicrobials. Among the 110 Watch group antibiotics, WHO EML enlisted 11 antibiotics as first or second choice of empiric treatment options. However, these antibiotics are recommended only for specific and limited indications. In addition, the Reserve group consists of antibiotics and antimicrobial classes for the treatment of MDR infections. Antibiotics in this group should be considered as “last resort” and also should be highly specific for patients and settings when all other antibiotics have failed. Till date, 22 antimicrobials have been classified as reserve group. In the recent global action plan, WHO enlisted seven reserve group antibiotics in the EML (191).

For both early and late onset neonatal sepsis, the most commonly recommended and used antibiotics, as per the WHO guidelines, is a β-lactam antibiotic (most commonly ampicillin) combined with an aminoglycoside (most commonly gentamicin) (191). Recently, in 2019, the use of AwaRe group of antibiotics was assessed in a pediatric survey across 56 countries (192). The study revealed that among the Access group of antibiotics, gentamicin and ampicillin were commonly given to the septicemic neonates in most of the countries, including Africa, America, Eastern Mediterranean, Europe, and South-East Asia. However, in the Western Pacific region, amoxicillin and β-lactamase inhibitor were used as empiric treatment for neonates. In addition, the study also showed that among the Watch group of antibiotics, globally meropenem and/or cefotaxime were prescribed to the neonates suffering from bacterial sepsis. Moreover, in South-East Asia, among the Reserve group of antibiotics, colistin was given to the critically ill neonates (192).

Due to the emergence and spread of CRKP strains in the healthcare settings, the treatment options for neonates are narrowing. Added to this, are the challenges in sepsis diagnosis. This alarming scenario demands new antimicrobials or additional alternatives for the treatment of CRKP-infected septicemic neonates. Although currently there is a scarcity of new antimicrobials globally, combination therapy is an alternative. Such therapies can expand the spectrum of antibiotic coverage and synergism between the antibiotics with enhanced killing effect. In addition, new alternatives have also been suggested for CRKP infections though all may not be suitable for neonates. For example, avibactam (a non-β-lactam β-lactamase inhibitor) showed high effectivity against KPC- and OXA-48-producing CRKP strains when combined with ceftazidime (193). Similarly, other studies revealed that vaborbactam (a boronic acid derivative) when combined with meropenem can exert bactericidal activity against the KPC-2-producing CRKP strains (194). Current studies have also argued that plazomicin (a novel semisynthetic aminoglycoside) showed impressive activity against most of the CRKP strains (195). The use of polymyxins (including both colistin and polymyxin B) and fosfomycin have also been documented in several case studies for the treatment of CRKP infections in the neonates (196, 197). In the current AwaRe classification, WHO has enlisted these antibiotics in the reserve group for which significant clinical data are still missing (191).

In response to the critical priority pathogens, recently WHO has published a third review of the clinical antibacterial pipeline where they enlisted a total of 8 new antibiotics that gained market authorization since July 2017 (198). Of them, two β-lactam and β-lactamase inhibitor combinations (vaborbactam + meropenem and relebactam + imipenem), one aminoglycoside (plazomicin), and one tetracycline (eravacycline) were found to be active against the CRE. Although all of these newly launched antibiotics are active against the Class A and Class D carbapenemases but found ineffective against the MBLs. To combat the MBL-producing CRE, WHO has also enlisted several other antimicrobial agents that are now in clinical trials (Phase 1–3), such as cefiderocol (Phase 3), taniborbactam + cefepime (Phase 3), BOS-288 (Phase 2), zidebactam + cefepime (Phase 1), and nacubactam + meropenem (Phase 1) (198). All of these currently developed antibiotics and/or antibiotic combinations are active against the CRE but the clinical data regarding their effectiveness against neonatal septicemia are yet to come. Although several clinical trials are ongoing for neonatal sepsis, additional large-scale clinical trials, strict infection control measures, and antimicrobial stewardship programmes should be undertaken in future to treat infections caused by drug-resistant and virulent *K. pneumoniae* to arrest their further dissemination.

## Alternative Combat Approaches

The gut of a term neonate having normal-body-weight is mainly comprised of *Bifidobacterium spp., Bacteroides, Clostridium spp*. and *Lactobacillus spp*. (in minor proportion) (160, 199, 200) and serves key role in providing nutrients, providing defense against gut colonization by pathogens, and development of immunity of the neonate (160). Whereas, in preterm and/or low-birth-weight neonates, intestinal flora lacks colonization by the above mentioned favorable microorganisms (199). Preterm and/or low-birth-weight neonates are often colonized by Gram-negative bacilli, some of which can be opportunistic pathogens and may trigger an inflammatory response which plays the key role in the initiation of the necrotizing enterocolitis and sepsis (160, 200). If the neonates are given supplements of probiotics (consisting of bacteria which forms the healthy gut flora of term neonates) with human breast milk, it helps in reduction of inflammation (200) as well as translocation of pathogenic bacteria (160). The use of *Bifidobacterium spp*. and *Lactobacillus spp*, in different probiotic doses has shown significant decrease in necrotizing enterocolitis and neonatal death (199–201). Despite pre-existing skepticism that probiotics may increase sepsis instead of decreasing it (200, 202), systemic reviews and meta analyses of randomized control trials of large sample sizes have shown that probiotics indeed play a beneficial role in decreasing the rate of LOS in preterm low-birth-weight neonates (203–205). The bacterial strains used in the randomized control trials were mainly *Bifidobacterium bifidum, B. lactis, B. breve, B. infantis, Lactobacillus acidophilus, L. rhamnosus, L. reuteri. Streptococcus thermophilus* in varied doses (203). As probiotics not only provide a low cost, non-invasive, safer way to replenish the preterm neonatal gut with natural gut flora of a healthy term neonate, but also provides defense against LOS, it can be considered for routine use in LMICs where the load of LOS is overwhelming.

Preterm neonates of <32 weeks of gestational age lack maternal transplacental immunoglobulin; transfer of which, from mother to fetus, occurs only after 32 weeks of gestation. In the search for suitable alternative therapy for sepsis in preterm neonates, studies were conducted by administering intravenous immunoglobulin, granulocytes, granulocyte colony stimulating factor and granulocyte-macrophage colony stimulating factor, and pentoxifylline to neonates. The results were not promising except for pentoxifylline which caused decrease in all-cause mortality (206, 207). However, an monoclonal antibody (mAb)—Pagibaximab has shown some positive result (208). Recently, broadly reactive mAbs were raised against the capsular antigens of CRKP and those mAbs showed positive result in efficient killing of *K. pneumoniae* in mice model (209). Although, the efficacy of mAbs can be properly assessed only after clinical trials on neonates, but it shows some ray of hope in an otherwise grim situation.

## Conclusion

Antibiotic resistance in Gram-negative bacteria has clearly exposed that the World Health Organization (WHO) guidelines for the management of neonatal sepsis, which is currently ampicillin plus gentamicin, is in dire need of modification. The major burden of neonatal sepsis is borne by the LMICs where antibiotic resistance is high and microbiology laboratory support is inadequate. The number of documented neonatal infections caused by CRKP and hvKP are just the tip of the iceberg. Most cases are not recorded as the microbiology laboratories in resource-limited countries lack infrastructure and capability. This is probably likelier for hvKP, as these strains are not specifically detected in clinical microbiology laboratories. This lack of information is thus translated into treatment protocols. Studies have shown that the most severe forms of neonatal sepsis with an unfavorable outcome were due to virulent strains of *K. pneumoniae* (5). The clinician is however unaware of the hvKP phenotype. Some carbapenem resistance genes such as *bla*_KPC_ and *bla*_OXA−48_ are also difficult to recognize by routine disc diffusion tests. The presence of these genes may not be detectable by such tests as their capability to hydrolyze carbapenems are variable.

A recurring premise about carbapenemases is their ability to hydrolyze most β-lactam antibiotics, emergence of variants and their promiscuous nature. Plasmids are numerous and have facilitated intra and interspecies transmission of these genes particularly under antibiotic pressure. Globalization has encouraged their spread and most genes have within a few years of identification crossed boundaries and invaded new terrains. Clonal spread is uncommon but not unknown. The evolution of variants of enzymes that are more efficient or have better stability have also made them difficult to contain. Plasmids carrying carbapenem-resistant genes also carry other resistant genes in a bid to make a panel of antibiotics ineffective. With new carbapenem resistance genes being identified and new variants evolving, the problem of CRKP and hvKP that we recognize presently is incomplete.

Another overlooked aspect of *K. pneumoniae* is that it commonly resides in the human gut. The rate of colonization of *K. pneumoniae* increases drastically in hospitalized patients with invasive devices, antibiotic exposure, and prolonged stay. Needless to say, that this would also happen with premature, low-birth-weight neonates with a pristine gut. *K. pneumoniae* that colonize the gut in the hospital are resistant to antibiotics and the gut provides an environment apt for the exchange of resistance genes. Studies have shown that such exchange of resistance genes frequently happen in the gut (210). Further, bacteria from the gut can translocate and cause sepsis in neonates. This can happen in neonates who have an immature immune system, lower levels of mucus and gastric acid production. The bacteria evade the gut barrier and cause sepsis. Our study on the neonatal flora showed that *K. pneumoniae* was not only the predominant organism that colonized the neonatal gut, but also the most common organism isolated from the neonatal blood specimens (162). The exchange of carbapenem resistance genes in the gut and subsequent translocation can further complicate the situation.

It is difficult to change things overnight and the economic implications of these changes may also be a constraint on health systems that are poorly funded. The COVID-19 situation has further exposed the fragility of health systems around the world and newborns have also been affected. Measures that can reduce infection rates can also reduce CRKP and hvKP. These include surveillance systems to recognize changes in etiology and drug resistance profile, improved laboratories for better and timely detection of pathogenic strains, setting empirical treatment guidelines based on the profiles and proper education of mothers and healthcare workers regarding sanitation. We have come a long way from the days when we understood microbes through the lens of a microscope. We now seem to have an unprecedented power over them by being able to understand their genetic make-up to the last nucleotide. Whole genome sequencing has opened up newer avenues to understand the bacterial genomes. This enormous amount of information generated can be honed to create new cures and products. The challenge is big but there is light at the end of the tunnel.

## Author Contributions

SB: conception, design, wrote the introduction, and discussion. SMu and SMi: performed literature search, wrote the initial draft, and prepared all the tables and figures. SB and SD: critically revised the manuscript. All authors read and approved the final manuscript.

## Conflict of Interest

The authors declare that the research was conducted in the absence of any commercial or financial relationships that could be construed as a potential conflict of interest.
